# A mixed-methods investigation of parent-child posttrauma discussion and the effects of encouraging engagement

**DOI:** 10.1080/20008198.2019.1644127

**Published:** 2019-07-29

**Authors:** Rosie McGuire, Rachel M. Hiller, Vanessa Cobham, Katharina Haag, Sarah L. Halligan

**Affiliations:** a Department of Psychology, University of Bath, UK; b School of Psychology, The University of Queensland, Brisbane, Australia; c Children’s Health QLD, Child and Youth Mental Health Service, Brisbane, Australia; d Department of Psychiatry, University of Cape Town, Rondebosch, South Africa

**Keywords:** Mixed-methods, parenting, child, trauma, support, family, PTSD, post-traumatic stress, posttraumatic stress, intervention, métodos mixtos, Crianza de los hijos, niño, Trauma, Apoyo, Familia, TEPT, Estrés postraumático, intervención, 混合方法, 教养方式, 儿童, 创伤, 支持, 家庭, 创伤后应激障碍, 创伤后应激, 干预

## Abstract

Recent developments in the child trauma field include preventative interventions that focus on augmenting parental support. However, we have limited knowledge of how parents experience trauma conversations with children. We examined how parents and children experienced both spontaneous trauma conversations and a structured task in which they generated a joint trauma narrative, following the child’s experience of an acute trauma. Parent and child ratings of distress during the structured narrative were low for all 127 families that took part, with child ratings of distress being lower overall than parent ratings. Task-related distress was positively associated with parent and child PTSD symptoms. Thematic analysis of semi-structured interviews conducted with a subset of twenty parents identified both facilitators of (e.g. open and honest relationship with child) and barriers to (e.g. parent/child avoidance of discussion) spontaneous trauma-related conversations with their child. Additionally, parents described the structured trauma narrative task as an opportunity to start the conversation with their child, to understand their child’s feelings, and for the child to process the trauma. However, the task was also uncomfortable or upsetting for some parents/children, and resulted in parents becoming more overprotective. The findings can inform development of low-dose interventions that encourage families to engage in trauma-related conversations following child experiences of trauma.

## HIGHLIGHTS

• Overall, parents and their children reported low distress levels when discussing the child’s trauma, although both parent and child were more distressed if the child or parent had high PTSS.• Parents reported facilitators (e.g. open and honest relationship) and barriers (e.g. avoidance of discussion) to spontaneous trauma-related conversations with their child.• Most parents considered a prescribed trauma-related discussion with their child as beneficial. • Results suggest a low-dose intervention encouraging families to discuss trauma would be well-received.

## Abstract (es)

Los desarrollos recientes en el campo del trauma infantil incluyen intervenciones preventivas que se centran en aumentar el apoyo de los padres. Sin embargo, tenemos un conocimiento limitado de cómo los padres experimentan las conversaciones de trauma con los niños. Examinamos cómo los padres y los niños experimentaron las conversaciones espontáneas sobre traumas y una tarea estructurada en la que generaron una narrativa conjunta sobre el trauma, siguiendo la experiencia del niño sobre un trauma agudo. Las calificaciones del malestar de los padres y los hijos durante la narrativa estructurada fueron bajas para las 127 familias que participaron, y las calificaciones del malestar de los niños fueron más bajas que las calificaciones de los padres. El malestar relacionado con la tarea se asoció positivamente con los síntomas de TEPT de los padres y de los hijos. El análisis temático de las entrevistas semiestructuradas realizadas con un subconjunto de veinte padres identificó tanto a los facilitadores (por ejemplo, una relación abierta y honesta con el niño) como a las barreras (por ejemplo, evitar la discusión entre los padres y los hijos) de las conversaciones espontáneas relacionadas con el trauma con su hijo. Además, los padres describieron la tarea narrativa del trauma estructurado como una oportunidad para iniciar la conversación con su hijo, para comprender los sentimientos de su hijo, y para que el niño procese el trauma. Sin embargo, la tarea también fue incómoda o molesta para algunos padres/hijos, y resultó en que los padres se volvieran más sobreprotectores. Los hallazgos pueden informar el desarrollo de intervenciones de dosis bajas que alientan a las familias a participar en conversaciones relacionadas con el trauma después de las experiencias de trauma del niño.

## Abstract (zh)

儿童创伤领域的最新发展之一是预防性干预，其关注点在于增加父母的支持。然而，我们对父母如何与孩子进行创伤对话的认识有限。我们调查了父母和孩子如何自发进行有关创伤的谈话。同时在一项结构化任务中，我们考察了他们如何在孩子的一次急性创伤经历后，共同叙述这个创伤故事。在全部的127个参与家庭的结构化叙事中，父母和孩子对于痛苦的评分程都很低，其中儿童对痛苦的评分总体上低于父母。和任务相关的痛苦与父母和儿童的创伤后应激障碍的症状呈正相关。随后对其中20名父母进行的半结构式访谈，主题分析的结果揭示了与孩子自发进行创伤相关的谈话的辅助因素（例如与孩子公开、坦诚的关系）和妨碍因素（例如父母或孩子逃避讨论）。此外，父母将结构化创伤叙述任务描述为一个可以与孩子开始对话，了解孩子的感受以及让孩子处理创伤的机会。然而，对于一些父母或孩子来说，这项任务也令他们感到不舒服或烦恼，导致父母变得更加过度保护。本研究结果可为制定轻度干预措施提供指导，鼓励家人在儿童经历创伤后参与创伤相关的谈话中。

Exposure to single incident trauma, such as road traffic accidents and other accidental injury is common in childhood (Alisic, Van der Schoot, van Ginkel, & Kleber, 2008; Copeland, Keeler, Angold, & Costello, 2007). Following acute trauma exposure, children are at risk of a range of poor outcomes including posttraumatic stress disorder (PTSD). Large studies on the course of child PTSD show that while most young people are resilient to potentially traumatic experiences, showing either low symptoms overall or initial symptoms that quickly decline, a significant minority will show persistent or worsening PTSD symptoms (PTSS) (Le Brocque, Hendrikz, & Kenardy, 2009; Liang, Cheng, Zhou, & Liu, 2019). An estimated 10–20% of trauma exposed children develop chronic PTSD (Alisic et al., 2014; Hiller et al., 2016; Le Brocque et al., 2009), and an even higher proportion of children will experience elevated sub-clinical PTSS, which can be associated with functional impairment and distress at a comparable level to those who meet full diagnostic criteria (Carrion, Weems, Ray, & Reiss, 2002).

One factor implicated in the development of posttrauma distress is social support, with a meta-analytic review showing that children who perceive better social support experience less distress (Trickey, Siddaway, Meiser-Stedman, Serpell, & Field, 2012). For children, parents are a key source of informal support following trauma exposure (Williamson, Creswell, Butler, Christie, & Halligan, 2017). Recent work that recorded daily conversations of children exposed to acute trauma found that families initially spent an average of 46 minutes per day discussing the child’s injury and the emotions associated with it (Alisic et al., 2017). Other evidence indicates that too much or too little trauma talk can potentially lead to higher levels of child distress posttrauma (Stein et al., 2004; Wilson, Lengua, Meltzoff, & Smith, 2010). In terms of the content of parent-child trauma discussion, a parent’s focus on threat or the child’s vulnerability, overprotection, and encouragement of avoidant coping have all been associated with higher child posttrauma distress (Ehlers, Mayou, & Bryant, 2003; Hiller et al., 2018). Conversely, parents can use such conversations to model positive coping strategies and discuss emotions, thus helping the child to process their traumatic experiences adaptively (Alisic et al., 2017; Hiller et al., 2018; Marsac, Donlon, Winston, & Kassam‐Adams, 2013; Sales & Fivush, 2005).

Taken together, research suggests that parents frequently talk to their children about trauma, and that the trauma-focused support they provide can influence child adjustment (Alisic et al., 2017; Hiller et al., 2018). Nonetheless, there are a range of potential barriers that may prevent parents from providing effective support for their children posttrauma. In particular, parental distress may limit their own emotional capacity, or children may perceive that their parent is too upset to engage in discussion, each of which may prevent parent-child trauma talk (Gil‐Rivas & Kilmer, 2013). Providing support for this, a recent systematic review found evidence that parental PTSD can negatively influence the quality of the parent-child relationship, including more frequent use of controlling and hostile practices and higher levels of parenting stress (Christie, Hamilton-Giachritsis, Alves-Costa, Tomlinson, & Halligan, 2019). In addition to the potential role of their own distress, parents report having concerns about the possible emotional distress that trauma reminders may cause their child, and experience uncertainty about the best way to support their coping (Williamson, Creswell, Butler, Christie, & Halligan, 2016; Williamson, Creswell, Butler et al., 2017).

Evidence is accumulating with respect to the potential benefits of engaging parents in psychological interventions for children, with parent-focused interventions being increasingly utilised in the child mental health field (e.g. Brown et al., 2017; Cobham, Filus, & Sanders, 2017; Creswell et al., 2017). There has been growing interest in low-dose, parent-focused prevention/intervention programs in the context of child PTSS (Berkowitz, Stover, & Marans, 2011; Marsac, Kassam-Adams, Hildenbrand, Kohser, & Winston, 2010). As trauma-related conversations between parent and child would seem a necessary component of a parent-led approach, understanding how such conversations are experienced is important.

In the current study, we used a mixed-methods approach to explore parents’ views on engaging in discussions with their child about the child’s trauma. We were particularly interested in understanding factors that might facilitate or hinder whether a parent feels comfortable to initiate these discussions, and on how such conversations were experienced by parents. Parents and children were recruited within 1 month of the child’s acute trauma (typically a vehicle collision or other accidental injury), which led them to attend their local emergency department (ED). As part of study assessments, the parent was asked to engage their child in a discussion of the trauma. We obtained quantitative data relating to parent-child experiences of distress during this task. In addition, we completed qualitative interviews with a subset of parents approximately 3-months later, in which we explored parents’ experiences of spontaneous trauma conversation with their child in the weeks after ED attendance, and of engaging in this conversation within the context of the structured task.

## 1. Method

### 1.1. Participants and recruitment

Ethical approval was provided by the University of Bath Research Ethics Committee and the Oxford A NHS Research Ethics Committee. Participants came from a longitudinal study of 132 6–13 year olds and their parents who were recruited from EDs in the UK after exposure to acute trauma (predominantly road traffic accidents and other accidental injuries). Exclusion criteria were: the presence of a significant learning or neurodevelopmental disorder in the child that precluded mainstream schooling; injury resulting in a significant traumatic brain injury; suspicion of intentional injury by the child (self-harm) or the parent (maltreatment). ED staff first approached potentially eligible families and obtained their agreement for contact by a member of the research team. Informed consent and assent were then obtained from parent and child prior to their participation. Participants completed assessments at 1-month posttrauma and then 3-months and 6-months later. For full study recruitment procedures and assessments see Hiller et al. (2018).

Of the original 132 families, 127 parent-child pairs participated in a joint trauma narrative task at the 1-month assessment. For the qualitative component, we interviewed 20 parents approximately 3-months after the parent and child had participated in the task (i.e. at the 3-month follow-up assessment). An opportunity sampling method was used, with consecutive parents being invited to participate to coincide with the larger study assessment.

### 1.2. Child PTSD diagnosis measure

At the 1-month assessment, trained researchers administered the Anxiety Disorder Interview Schedule-PTSD Module (ADIS-PTSD; Silverman, Albano, & Barlow, 1996) to parents and children. The ADIS-PTSD is a well-validated diagnostic tool for PTSD, based on DSM-IV-TR criteria. Diagnostic inter-rater agreement was established on 25% of interviews (k = 1.00). The combined parent and child report was assessed to determine whether the criteria for a PTSD diagnosis was met (without the temporal requirement of >1 month), based on both a clinician severity rating and an interference rating of four and above.

### 1.3. Parent PTSS measure

At the 1 month assessment, parents completed the Posttraumatic Stress Diagnostic Scale (PDS), a widely used, self-report measure of PTSD (Foa, Cashman, Jaycox, & Perry, 1997). The PDS includes 17-items indexing symptoms of DSM-IV PTSD, and asks adults to rate each on a 0 (*not at all or only one time*) to 3 (*almost always*) scale, resulting in a possible total symptom score of 0 to 51. The strong internal consistency of the measure was replicated in the current study (α = .94).

### 1.4. Narrative task

As part of the initial assessment at 1-month posttrauma, parents were asked to engage their child in a conversation about the traumatic event (joint trauma narrative). They were instructed to begin just before the event happened and to include anything that they thought was important. There was no time limit and the task was video recorded for later quantitative coding (see Hiller et al., 2018). Following the free-report component, parents were provided with 13 prompt cards that gave them specific questions to ask about the child’s thoughts and feelings during and after the event, e.g. ‘*How did you feel when you were in the hospital?*’. Subsequently, parents and children each independently rated their distress (i.e. how upset they felt) during the conversation on a scale from 0 (*not at all*) to 10 (*very, very*).

### 1.5. Qualitative interviews

We used a semi-structured interview format to explore: (i) Parental experiences of spontaneous trauma discussions with their child prior to the task; (ii) Their experiences of engaging in the task (e.g. what it was like to have that conversation); and (iii) Potential consequences of the structured trauma conversation for how they understood or supported their child’s posttrauma reactions.

Questions were open-ended (e.g. ‘*How did you find having that chat?’*), with some specific prompts to clarify meaning or elicit further information (e.g. if they had already discussed the accident with their child before the research task, *‘what was that like? [How] was it different?’*). There was no time limit for the interview; they lasted between 3 minutes and 20 minutes, with a mean interview length of 8 minutes 43 seconds (SD = 4 minutes 36 seconds). All interviews were audiotaped and transcribed verbatim, with any identifying information removed during transcription.

### 1.6. Analysis

To understand how parents and children found engaging in conversation about the trauma, for the entire study sample (*N* = 127) we derived descriptive data from the distress ratings collected following the narrative task, and used independent samples *t*-tests to explore whether distress when talking about the trauma was associated with: parent/child sex; child age (6–9yo v 10–13yo); whether the parent witnessed their child’s trauma; and presence/absence of child PTSD. We also used bivariate correlation analyses to test whether parent and child task-related distress correlated with each other, and whether either was associated with parents’ own PTSS severity. Finally, we ran a paired samples *t-*test to compare parent and child ratings of distress. Average distress ratings from the subsample of 20 families that were later interviewed are also presented.

The 3-month transcribed interviews were imported to NVivo v10 for coding. Transcripts were coded by RM using an inductive approach to thematic analysis following the steps proposed by Braun and Clarke (2006). RM first read all transcripts to allow for data immersion, and then initial codes were systematically generated (Braun & Clarke, 2006). To ensure coding remained consistent, all transcripts were re-checked, with particular attention paid to earlier transcripts to ensure any relevant newer codes were not missed. The codes were then linked together when searching for and developing candidate themes. To ensure academic rigour and reliability, a second coder (KH) then read all of the transcripts and developed themes, blind to the original candidate themes. Following this, both coders compared their themes; they were very similar with almost identical quotes within each one, the key discrepancies were in theme titles and organisation. Between them, researchers revised these discrepancies by re-examining the data together until a consensus was met.

After themes were developed and revised, feedback regarding data interpretation and analysis was obtained from co-authors RH, SH and VC, who are experienced in child psychopathology research and qualitative methods. This resulted in the restructuring and combination of some subthemes, although the overall themes generated by the coders remained unchanged. We considered whether the presence of child PTSD might affect the parent’s experience of the conversation, by systematically examining the themes identified to check for clustering according to whether parents had children with or without PTSD. However, there were no patterns in the qualitative data suggesting that themes were different between these groups. Thus, themes are presented for the overall sample.

## 2. Results

### 2.1. Participants

There were 132 children recruited for the larger study, of whom 127 completed the joint narrative task (62.2% male), aged 6–13 years (*M* = 9.8, *SD *= 2.0), along with their participating parent, predominantly mothers (89.8%), aged 25–60 years (*M* = 39.9, *SD *= 6.9). A large proportion of these children (42%) were given a hospital triage rating of one, meaning they required immediate care. The majority of children had experienced a motor vehicle accident as their index trauma (52%). Other index traumas included serious accidental injury (30%; e.g. serious falls), acute medical episode (7%; e.g. acute anaphylaxis), assault (2%), or other event (9%; e.g. house fire, near drowning). At 1-month posttrauma, 26% (n = 34) of children met criteria for PTSD (without the temporal requirement, if <1 month post-trauma). Detailed sample information is presented in Hiller et al. (2018).

### 2.2. Distress during the joint trauma narrative

From the 127 parent-child pairs who completed the joint narrative task, parents rated their distress during the task (i.e. discussing the event) an average of 2.80 (*SD* = 2.63), from a possible 0–10 score. Thus, overall, parents experienced relatively low levels of distress when discussing the trauma with their child. Nonetheless, 30% of parents rated their distress as a five or above, showing a significant minority experienced elevated distress when recalling the event with their child. Children also reported overall low levels of distress when asked how they felt whilst recalling the event with their parent, *M* = 1.85, *SD *= 2.44. A paired-samples t-test showed children’s distress ratings were significantly lower than parents’ distress ratings, *t*(123) = 4.33, *p* < .001. Only 12% of children rated their distress discussing the event as five or higher on the 0–10 scale. Parent and child distress when talking about the trauma were moderately positively correlated (*r* = .57, *p* < .001).

We examined factors that might influence parent and child distress during the joint trauma narrative task. See Table 1 for a breakdown of parent and child distress scores by key demographic and trauma characteristics. We found no evidence that child age or sex was associated with distress in either parent or child, or that whether or not the parent witnessed/was involved in the event (versus learning about it) was associated with their distress when recalling the trauma. The distress ratings of children engaging in trauma-related conversation with their mother were significantly higher than children discussing the trauma with their father. Furthermore, both parent and child distress ratings were higher if the child met PTSD diagnostic criteria, than if they did not meet criteria (see Table 1). Similarly, parental PTSD symptoms were moderately positively correlated with both their own distress during the task (*r* = .57, *n* = 119, *p* < .001), and with their child’s distress (*r* = .32, n = 116, *p* = .001).

**Table 1.** Parent and child distress ratings (N = 127), reported by key demographic and trauma characteristics, with t-tests examining associations with narrative task distress ratings.

	Distress ratings during task	
Characteristic	Parent *M* (*SD*)	Child *M* (*SD*)
**Sex of child**		
Male (n = 79)	2.92 (2.62)	1.87 (2.61)
Female (n = 48)	2.60 (2.67)	1.81 (2.17)
	*t*(124) = .61, 95% CI [−.67, 1.25]	*t*(121) = −.12, 95% CI [−.91, .81]
**Sex of parent**		
Male (n = 13)	2.54 (1.90)	.92 (1.12)
Female (n = 114)	2.81 (2.71)	1.88 (2.42)
	*t*(124) = −.36, 95% CI [−1.81, 1.26]	*t*(28.2) = −2.48*, 95% CI [−1.75, −.17]
**Age of child**		
6–9 years (n = 55)	3.09 (2.86)	2.19 (2.72)
10–13 years (n = 72)	2.55 (2.45)	1.47 (1.95)
	*t*(124) = 1.15, 95% CI [−.39, 1.48]	*t*(90.1) = 1.63, 95% CI [−.16, 1.59]
**Parent witnessed trauma**		
Yes (n = 53)	3.08 (2.73)	2.20 (2.63)
No (n = 74)	2.61(2.56)	1.61 (2.29)
	*t* (125) = −.986, 95% CI [−1.41, .47]	*t* (122) = −1.33, 95% CI [−1.47, .29]
**Child PTSD diagnosis**		
Absent (n = 96)	2.29 (2.09)	1.34 (1.90)
Present (n = 31)	4.29 (3.49)	3.21 (3.00)
	*t*(37.2) = −3.01**, 95% CI [−3.34, −.65]	*t*(35.2) = −3.16**, 95% CI [−3.07, −.67]

*p < .05; **p < .01; ***p < .001

### 2.3. Qualitative analysis

Of the 20 families in the qualitative study subset, the majority of participating parents were mothers (only one father), and their average age was 38.8 years (*SD *= 7.7, Range = 26–60 years). Their children (14 male, 6 female) were on average nine years old (*SD *= 2.3, Range = 6–13 years). The most common trauma for this sub-group was a motor vehicle accident, either with the child as a passenger (15%) or a pedestrian (45%). Other traumas included serious accidental injury (25%), assault (5%), acute medical episode (5%), or other event (5%). Five of these 20 children (25%) met criteria for a PTSD diagnosis at the first assessment, around 1-month posttrauma. Parents and children in the qualitative subsample showed similar distress ratings during the narrative task to those of the main sample (parents *M* = 2.37, *SD =* 3.17; children *M* = 1.79, *SD *= 2.62). Therefore, the breakdown of characteristics within this sub-sample is similar to that of the larger sample.

From the analysis of the 20 interviews, parents highlighted three key themes when reflecting on their experience of trauma-related discussion. Parents gave reasons for engagement or non-engagement in spontaneous trauma talk with their child, describing facilitators (Theme 1) and barriers (Theme 2) to their initial posttrauma discussions. Parents also reported on their experiences of the joint narrative task, and the impact it had on them and their children afterwards (Theme 3).

### 2.4. Theme 1: facilitators of spontaneous trauma talk between parent and child

The majority of parents (15/20) had spontaneously discussed the trauma with their child to some extent in the immediate aftermath, and a number of facilitators of such conversations emerged from the data.

#### 2.4.1. Subtheme 1: passive factors – parents perceived that an honest and open relationship with their child meant the child could initiate discussions

The majority of parents who said they had already engaged in posttrauma discussions about the event with their child put this down to having a good relationship with them, in which both parent and child were honest and open with each other about their thoughts and feelings. This meant that a forum for discussing the event was already established and both parties felt comfortable enough to approach the topic.

Participant E: *I put it down to our relationship and the fact there’s nothing hidden about the accident or any of that.*

One parent spoke about adopting their own mother’s parenting style as they thought it was successful in creating an open relationship, which encouraged mature conversations. This parent wanted to do the same for their child so that they would feel comfortable expressing themselves.

Participant D: … *my mum did the same with me, you know talked openly as if I was an adult … I felt included and my ideas and what I had to say was valid … I’ve tried to do the same with her so she isn’t scared to say something if she’s sad or happy or something made her angry, you know she is able to say something.*

Many parents that identified as having a good relationship with their child also reported the belief that their child was comfortable enough to approach them to discuss their feelings. Consequently, parents often thought it best to let the child initiate posttrauma discussions as and when they were ready.

Participant B: *It’s up to him really whether he comes to me or his dad. He’s quite open.*

Participant R: … *she’s quite an open child she will let you know if something’s bothering her.*

It is interesting to note that participant R was one of the few parents who had not previously spoken with their child about the trauma before they participated in the joint narrative task. This may have been because the young person was not bothered by the event and did not feel the need to discuss it, but could also show that leaving it to the child to initiate the conversation could mean the discussion never occurs, even if it may have been beneficial.

#### 2.4.2. Subtheme 2: active factors – parents actively encouraged the child to discuss their experience

Other parents actively encouraged their children to engage in trauma-related discussion by creating opportunities for them to talk about it, rather than waiting for their child to initiate the conversation. This included directly asking their child how they felt about the traumatic event and explicitly making time to listen to what their child may be disclosing.

Participant G: *… I would ask [child] how he was feeling right from the start and what he thought about the incident.*

Participant T: *… I think it’s really helpful to occasionally just stop and listen to your child really intensely about something …*

One parent emphasised that they intentionally used posttrauma discussion with their child to develop strategies to cope with the current trauma, resulting in the child having coping tools that could also be used for adverse events in the future.

Participant S: … *we talked about having like a coping box in your head, one day it’s all gonna be in there etc so- it’ll be fine … we just discussed- you know what to do from now on …*

### 2.5. Theme 2: barriers to spontaneous trauma talk between parent and child

Parents also identified factors that limited their desire or their ability to talk about the trauma with their child in the aftermath of the event.

#### 2.5.1. Subtheme 1: parent or child did not want to dwell on it or make a fuss

The most common reason given by parents for not engaging in posttrauma discussion was the belief that talking about it would result in unnecessarily dwelling on the traumatic event or making it more of a focus in a way that was seen as unhelpful. For some parents, this attitude was highlighted as a common coping strategy in everyday life.

Participant F: *I’m very much a … get it over and done with and let’s move on sort of thing … it’s like when they fall and have an accident … ‘Rub it better quickly and let’s get up and get on’ … I don’t sort of dwell too much.*

In other cases parents suggested that it was their child who would prefer to move on quickly and not dwell on the traumatic event by discussing it. In these cases parents felt that their child was unaffected by the traumatic event because they did not show any obvious signs of distress. Therefore parents did not encourage discussion, as it was assumed that the child was coping well.

Participant Q: *I think [child] is the kind of child that kind of just gets on with it … He doesn’t make a fuss … I never got the feeling- that he was- upset about it … I never got the feeling that there was a need to ask him …*

#### 2.5.2. Subtheme 2: parent avoided discussion of trauma due to distress

Another barrier reported by parents was that they themselves actively avoided posttrauma discussion. This was a common theme amongst the five parents who had not previously discussed the trauma before the joint narrative task; most of these parents emphatically stated they had not wanted to discuss the event with their child in the aftermath of the trauma. These parents’ responses frequently indicated significant trauma-related distress, resulting in a desire to avoid discussions about the traumatic event.

Participant H: *I think we avoided it because we weren’t sure – I think all of us were a bit unsure as to how we were all feeling we didn’t want to express it and I also avoid talking about it to be honest because it’s not something I want to look back on … For me even now I still try to avoid the thought about it.*

One parent also mentioned that they had avoided posttrauma discussion with their child because they themselves had not spoken to anyone else about the trauma. Consequently, they felt they needed support to process the trauma and reduce their distress before they could speak to their child about it, and perceived that they needed an outsider’s perspective to advise them on how to approach the situation.

Participant L: … *I hadn’t – sorted it out in my head … And I hadn’t talked to anybody about it … had someone had said ‘oh I think you need to do this’ or … ‘I can see you both need this’. Sometimes you need that other person to tell you and I just don’t have it.*

Protecting their child was also a reason given for avoidance of discussion by some parents, including some of those that had said they were too upset themselves to discuss the trauma. These parents said that they thought it would be too distressing for their child to discuss the event.

Participant H: *I’d have avoided it … to protect him … because I would feel that I didn’t want him to talk about it to make him feel unhappy.*

#### 2.5.3. Subtheme 3: child avoided discussion of trauma

Some parents reported that their child was actively avoiding posttrauma discussion. These children would evade any engagement in discussion from their parents or others; even being in ear-shot of a conversation about their trauma caused them to protest. Sometimes parents linked this to child distress, but other parents perceived active avoidance to be rooted in the child’s personality and did not attribute avoidance to more emotive reasons.

Participant A: *No cos he wouldn’t talk about it cos he was too scared and a bit shy he didn’t like to talk about it … he was a bit wary about talking about it. He didn’t like to, he’d tell people to shut up and not talk about it … I think it just upset him.*

A couple of parents discussed their own, visible, distress following their child’s trauma, which they believed to be the cause of the child’s active avoidance.

Participant T: *A child doesn’t want to upset their parents … he does realise that what he says affects me …*

#### 2.5.4. Subtheme 4: family too busy to sit down and talk about it together

More practical barriers to discussion were also mentioned, with parents saying they would have liked to sit down with their child to discuss the traumatic event, but that it was difficult to create the right environment for that opportunity due to having other siblings around, or being busy with work and other commitments.

Participant L: … *just really busy we don’t get around to it … being a single parent, having to manage the rest of their life and my job.*

### 2.6. Theme 3: parents’ experience of the joint trauma narrative, and its impact on the parent and child

Parents described both their ‘in the moment’ experience of having a structured, trauma-focused conversation with their child, and their perceptions of the potential consequences of that experience. Almost all parents (17/20) described at least one positive aspect of the experience (subthemes 1–3). However, almost half of the parents (9/20) also described at least one potentially negative aspect (subthemes 4 and 5).

#### 2.6.1. Subtheme 1: the task provided a structured opportunity for the child to process the trauma emotionally

Although most parents had already engaged in discussion about the event with their child prior to study participation, many said that the task was still helpful as the nature of the questions encouraged their child to talk more in depth about it than they had previously and to address their emotions. Parents perceived that it might have been particularly helpful in allowing their child to talk about any problems or more negative emotions, as they may not have had the opportunity or felt comfortable enough to discuss these spontaneously.

Participant J: … *it was fine I mean most of it I’d already spoken to him about anyway … I think it was just nice for him to be able to talk again and maybe having the option to kind of make him talk about it, rather than just let him talk when he felt like it.*

Other parents highlighted difficulties getting their child to speak about the trauma, due to generally limited conversation around difficult topics or more broadly. Being in a setting where they had to sit down and talk in a structured way for a period of time, meant that they had the opportunity to open up more than they usually would.

Participant Q: … *it was nice to- let him open up a little bit because he is the- thirteen year old lad that … will talk as much as he- generally has to … to get him to actually talk a little bit more was quite nice actually.*

Building upon this point of creating an opportunity to share, many parents suggested that by doing so, their children were able to process the traumatic event, get closure, and move on.

Participant D: … *she’s digested it and thought about it and now she can move on from it … it was discussed and she was included in the conversation … its helpful from that point of view I think, to get closure.*

Some parents said that it was good to use the joint narrative task, as well as other posttrauma discussions, to reassure their child that they are safe now and a similar traumatic incident is unlikely to happen again.

Participant C: … *we went through all of this because of what happened not because it’s a regular occurrence [child] and so by doing that task included with the whole study I think it helped [child] to realise that it isn’t likely to happen again …*

#### 2.6.2. Subtheme 2: the task gave parents the space, confidence and skills to start a conversation about the trauma

Some parents said that the main positive of completing the joint narrative task was that it ‘forced’ them to take the time to sit down with their child to discuss the trauma. This seemed to be particularly true for parents who had previously reported practical obstacles (e.g. insufficient time) after the trauma as a key barrier for initiating such conversations.

Participant J: … *having somebody say ‘right sit down and have this chat with him’ … is a good thing because whether I would have done it off my own back- actually sat down with him on his own and talk to him like we did so it was quite a nice thing to do … It was a very busy house so it was quite nice to have that opportunity really to just sit with him.*

For the few parents who had previously actively avoided posttrauma discussion, the joint narrative task served as an ‘ice-breaker’ to begin trauma-related conversation. They reported that discussing the event was not as bad as they anticipated and they realised their child actually wanted to have that discussion. This led to parents approaching the topic more openly, and in some cases actively encouraging future discussions.

Participant K: *… I think it helped me to tell [child] that he could talk about it when he wanted to … He wouldn’t talk about it cos he knew I didn’t want to but … He knows now that he can talk about it … You know we talked about it now so.*

Parents also described learning more about how to handle approaching a sensitive topic such as trauma through completing the joint narrative task. Many said that they found the question prompts useful, and that in the future they would use similar questions to prompt their child to talk about things.

Participant G: *… it flagged up more questions that I could have asked him at the time of the accident … it helped me to think ‘ooh actually in future events I could be asking more sort of questions’ …*

#### 2.6.3. Subtheme 3: the task improved parents’ understanding of the child’s thoughts and feelings

Parents reported that the task allowed them to see things from their child’s perspective more, as it involved co-constructing a narrative and sharing how they felt. This gave parents a greater insight into their child’s experience of the trauma, their feelings towards it, and its influence on their behaviour.

Participant O: … *really- helpful- useful … to see it from her perspective, we’ve never spoke about it- before … in that detail.*

Participant B: *I think I just listened to him more … to understand how he was feeling in it like there’s my feelings towards it … And obviously there’s his as well but for him to actually go through that and for me to see that it’s two completely different opinions …*

Many parents also described how the task gave them the opportunity to check up on their child in a more structured way. Providing their child with this clear opportunity to raise concerns, and having the opportunity to ask in-depth questions about their feelings, allowed parents to obtain often wished-for feedback that their child was coping well, which they perceived as beneficial.

Participant H: *… it made me feel better about the situation as well I suppose … I knew he wasn’t feeling so bad about it all … it made me feel able to cope with it a bit more … it made me feel happy the fact that he is okay with everything and he’s moving on so it did help in a big way.*

Becoming more aware of their child’s feelings following the task encouraged some parents to check up on their child more often. After creating the narrative, many were also more aware of potential triggers of negative memories, and despite often being unable to avoid them, they could ensure the child was okay when confronted with these triggers.

Participant J: *Yeah watching out for how he is feeling … yeah I haven’t stopped taking him anywhere that would remind him of it – he needs to sort of just learn how to cope with the feelings … But yeah, it definitely made me sort of check in on him a bit more.*

#### 2.6.4. Subtheme 4: discussing the trauma during the task was uncomfortable or upsetting for the parent and/or the child

For some parents, the joint narrative task was identified as a somewhat negative experience as they were not comfortable discussing their feelings, particularly in relation to the trauma. These parents reported feeling uncomfortable when recalling the event with their child, possibly because they had been avoiding thinking and talking about it before.

Participant B: *Well I’m a person who don’t really like to talk about how I’m feeling*.

Participant F: *I was a little bit uncomfortable but it wasn’t too bad – just kind of going through the event again with her.*

One parent, who was visibly distressed during the joint narrative task, said that this was due to some of the question prompts. While the parent had already previously discussed the event with their child, the task was the first time that they had been encouraged to discuss how they felt about the trauma, rather than only the practicalities of what had happened.

Participant F: *I’d been able to talk about the facts really easily but I hadn’t been able to let the emotions come out and so those questions did prompt some of those emotions to come out which is why I got upset.*

Some parents suggested they were concerned that the joint narrative task induced anxiety or distress in their child as it made them revisit the trauma. Many of these parents had not previously discussed the trauma with their child for this reason.

Participant P: … *I was worried- that it would actually- almost give [child] more anxiety … In some ways it was a negative in that sense, it sort of brought it right back to the front of his mind to dwell on – but then again it’s never good to keep emotions down.*

#### 2.6.5. Subtheme 5: parent more overprotective of their child after the task

Several parents reported being more protective of their child after discussing the trauma in the joint narrative task. This was either due to the child revealing that they were upset in a way that the parent had not realised before, or just because discussing it ignited some worry or distress within the parent, which led parents to perceive their child as more vulnerable.

Participant F: *I think from that from that chat we had I thought she was further along than what she is.*

For some parents, this resulted in an overprotective approach of keeping their child away from any trauma reminders or potential future accidents.

Participant A: *I was very like uh protective (laughs)*

Interviewer: *So do you think you’re more protective after having that conversation?*

Participant A: *Yeah definitely … I wouldn’t let him out (laughs) I don’t like letting him out …*

Other parents did not prevent their child from doing things, but instead made sure they were aware that they needed to be more careful in certain situations, in order to prevent re-exposure to a similar trauma.

Participant S: … *I made sure – that when it’s icy and stuff like that, it’s made me a bit more aware … And you know just to reiterate in the morning ‘no it’s a bit icy so the drivers and yourself won’t be able to stop as quick’.*

## 3. Discussion

We used mixed-methods to explore parents’ experiences of both spontaneous and prescribed posttrauma discussion with their child. Overall, qualitative findings suggest that parents hold broadly positive views about engaging in trauma-related conversations with their children, as an opportunity to develop shared understanding and provide support. This was true with respect to both spontaneous trauma talk and the structured narrative task, for which both parents and children reported low mean levels of associated distress. At the same time, barriers to spontaneous trauma discussions were also described, particularly relating to parental uncertainty and parent/child distress, and some parents found the structured narrative provoked distress and overprotective parenting. Task distress ratings were higher where children and parents reported higher PTSD.

The majority of parents in our study described spontaneously engaging in trauma-focused conversations with their children as a positive way to facilitate child coping, consistent with previous evidence suggesting that in the aftermath of an acute event, trauma talk is common for many families (Alisic et al., 2017; Williamson et al., 2016). This largely positive view of trauma conversations also extended to the structured narrative task, which was perceived as creating an opportunity for the child to engage in a conversation about their trauma if they had not already, or to talk more deeply about their emotions, helping to process their reactions. Parents described gaining a better understanding of their child’s posttrauma emotional reactions and needs through the narrative task, and found that it provided an opportunity to reassure their child that they are safe, and to receive reassurance themselves if their child is coping well. These findings suggest potential benefits of facilitating parent-child trauma talk, particularly in terms of increasing parental understanding, which is an important first step in providing support (Alisic, Boeije, Jongmans, & Kleber, 2012; Carpenter et al., 2017; Pynoos & Nader, 1988).

Our study also highlighted variation in how parents approached trauma conversations with their child, which was related to perceived child characteristics. Parents who said their child was less forthcoming about the trauma frequently took active steps to encourage discussion to find out how their child felt, whereas parents took a more passive role if they perceived their child to be generally open and forthcoming, allowing their child to initiate conversation. Waiting for the child to take the lead meant that in some cases, potentially useful conversations between parent and child did not take place. This may be unproblematic if children are coping well, but may be a missed opportunity for parents who are not always aware of the full extent of their child’s distress (Meiser-Stedman, Smith, Glucksman, Yule, & Dalgleish, 2007).

Other barriers to trauma-related discussion were also identified; these particularly centred on parents’ own distress, and their concerns about potential child distress, consistent with previous research (Alisic et al., 2012; Williamson et al., 2016). Some parents also suggested that their child had avoided discussing the trauma, as they were aware of their parents’ trauma-related distress. Despite parental concerns, overall distress ratings for the structured trauma narrative task were low for both parents and children, and children reported significantly lower mean distress during the discussion than their parents. This may be important information to share with parents. That is, that their own feelings of distress or being uncomfortable with trauma conversations, does not necessarily mean their child is also finding the conversation distressing. Indeed, where the joint narrative task was the first time the parent had talked to their child about the trauma, many reported that it also made them aware that the child had actually wanted to have this conversation, despite the parent’s own hesitations. That said, parent and child PTSS were both, perhaps unsurprisingly, significantly associated with higher reported distress when talking about the trauma. There was also a significant moderate correlation between parent and child distress during the task, potentially reflecting well-documented evidence of a moderate association between parent and child PTSS (e.g. Landolt, Ystrom, Sennhauser, Gnehm, & Vollrath, 2012). These sensitivities between parent and child, and their avoidance of discussing the trauma have been associated with greater child PTSS in previous quantitative work (e.g. Carpenter et al., 2017; Gil‐Rivas & Kilmer, 2013).

Given the duality of parent and child distress, providing more support for parents or their children with high levels of PTSS, who may be finding trauma-related communication difficult, could be particularly beneficial. Of note, whether or not the parent was involved in or witnessed the event, versus hearing about it, was not significantly associated with their later distress. There is mixed evidence about the role of parents’ direct involvement in children’s trauma and subsequent risk of parental PTSD (e.g. de Vries et al., 1999; Hiller et al., 2015). Given the lack of consensus, parents should be offered support regardless of whether or not they were directly involved in the trauma, particularly given parental posttrauma distress can have a direct effect on the child’s emotional wellbeing and broader family functioning (Landolt, Buechel, & Latal, 2011).

Parents in our study also identified uncertainties about whether it is more beneficial to approach or avoid trauma-related conversations and how to begin discussing such sensitive topics as being obstacles to trauma talk. For the parents that felt more distressed and unsure about having a trauma-related discussion, being encouraged to talk and having a structure to help them approach the topic allowed them to ‘break the ice’, which facilitated further subsequent discussions. Although many parents in our study had previously talked about the trauma with their child, for a proportion, the narrative task was their first conversation. Moreover, even for parents who reported previous trauma discussions, the structured conversation was perceived as being qualitatively different, providing new insights, particularly around thoughts and feelings, and opportunities to provide support. At the same time, it is important to note that negative aspects of the joint narrative task were also highlighted by some parents, who perceived it to be uncomfortable or distressing for themselves or their child, or felt more overprotective of their child after completing the task. The latter is a potential concern that should be addressed, given observations of associations between parental overprotection and child PTSS (Williamson, Creswell, Fearon et al., 2017).

These findings should be considered in light of some limitations. First, only 13 parents in the larger study were fathers, and only one parent in the qualitative component was a father. As such, our findings mainly represent the views of mothers. There was some evidence that child distress was lower during conversations with fathers, although the small number of study fathers means further research is needed to provide more reliable information about this possible effect. Second, the vast majority of the sample were involved in accidental traumas (e.g. car accident, sporting accident). Findings cannot be generalised to conversations following interpersonal trauma, where severe PTSD is more likely and/or levels of discomfort during conversations may be higher. This is particularly important considering that we found increased distress associated with trauma discussion where PTSS was present in either parent or child. Study of higher risk trauma exposed groups is essential.

In sum, our findings provide insight in to how structured support could help parents feel more comfortable engaging in trauma-related conversations with their child and improve parental insight into child coping. Whilst further study is needed to understand the potential benefits of such an approach for parent or child adjustment, parents reported on the positive impact that structured trauma conversations could have on the support they provide their child going forwards. This information, along with observations relating to why parents may or may not feel comfortable talking to their child about the trauma, are of potential importance when designing low-dose, parent-led interventions, which would most likely include the encouragement of conversations between parent and child. A first-line, cost-effective parent-delivered CBT has already been developed for children with anxiety (Creswell et al., 2017), with the majority (60%) of treatment gains maintained at a three to five year follow-up (Brown et al., 2017). In the PTSD field, parent-focused interventions also show significant promise for children exposed to acute trauma (Berkowitz et al., 2011). Our study suggests that structured intervention could equip parents with the skills and confidence to initiate trauma-related conversations with their child, and educate them on the potential advantages of this approach.
